# Analysis and Suppression of Crosstalk Stray Light in a Microlens Array Scanning and Searching System

**DOI:** 10.3390/mi14020336

**Published:** 2023-01-28

**Authors:** Zhiyang Lv, Yunhan Huang, Zhiying Liu

**Affiliations:** School of Opto-Electronic Engineering, Key Laboratory of Optoelectronic Measurement and Optical Information Transmission Technology of Ministry of Education, Changchun University of Science and Technology, Changchun 130000, China

**Keywords:** microlens array, beam crosstalk, stop array, stray light, optical simulation

## Abstract

The microlens array (MLA) system can aid in realizing fast beam deflection owing to the lateral displacement between arrays. The MLA system has the advantages of miniaturization and good functionality. However, during system operation, crosstalk beams are generated between each microlens array unit, introducing additional stray light, thus affecting the imaging contrast of the system. Therefore, this study uses the matrix operation method to trace the paraxial ray to trace the optical system and analyzes the generation mechanism of crosstalk stray light in the MLA system. Furthermore, this study proposes a crosstalk suppression method based on a stop array to reasonably suppress stray light. Finally, an example of an infrared array scanning infrared optical system is considered so as to verify the correctness and feasibility of the proposed crosstalk stray light suppression method. Therefore, this paper introduces the stray light suppression principle to guide the optical design process of the system, providing a theoretical basis for the design and analysis of the microlens array scanning and search system.

## 1. Introduction

The microlens array is widely used in optical imaging detection, laser radar, satellite remote sensing, and military fields because of its low beam-pointing deviation, high scanning rate, and rapid steering during dynamic imaging [1,2].

The processing technology of microlens is also developing with the demand. In 2016, researchers presented a method for preparing well-defined microlenses based on polymer phase separation in the presence of supercritical carbon dioxide (scCO2). Microlenses with dimensions from 2 to 15 μm and contact angles from 55° to 112° were successfully obtained through adjustment of the kinetic conditions and outgassing rate, affording great promise for applications in bioimaging, photolithography, light harvesting, and optical nanosensing [3]. In 2019, researchers proposed a manufacturing technique named laser catapulting (LCP), which enables the preparation of microlenses with controlled geometry and curvature. Arrays of circular, triangular, and cylindrical microlenses with a radius between 50–250 µm and 100% fill-factor can. Be obtained [4]. In 2021, a highly integrated planar annular microelectrode array was proposed to achieve an electrowetting tunable MLA. The planar microelectrode was fabricated by electrohydrodynamic jet (E-jet) printing and the liquid microlens was then deposited in situ on the microelectrode. This method can be beneficial for cell imaging, optofluidic systems, and microfluidic chips [5].

Several studies have been conducted in these areas. The University of Konstanz in Germany developed a micro-opto-electro-mechanical multiplexer system for fast imaging [6]. The University of Tokyo in Japan used piezoelectric ceramics to drive microlens arrays for beam scanning [7]. The Huazhong University of Science and Technology combined microlens array and convergent lens for beam deflection [8]. Tianjin University designed a microlens array system by integrating the transmitter and receiver for continuous scanning imaging [9]. However, with increasing the scanning angle, beam crosstalk occurs between the microlens array units; that is, light is incident on the adjacent lens units, interfering with the normal light transmission as stray light, thus reducing the imaging quality of the system.

Several solutions have been proposed to solve the problem of crosstalk between lenses. In 1993, Watson realized crosstalk-free and large fill factor beam control by adding microlens arrays that function as field mirrors, effectively expanding the radial displacement and improving the scanning field of view [10]. In 2004, researchers designed the second array as a double-sided lens array that consists of the objective and collimating lens arrays on the same substrate, so that the first side of the second row can be used as the field mirror, and they can move in the same manner; furthermore, a certain amount of eccentricity exists without generating stray light [11]. However, it is necessary to ensure that the front surface of the second array contains the image point of the first array. In 2018, a laboratory in Beijing proposed an imaging method combining microlens and stop arrays, enabling the microlens array to image in the staring field of view with a limited object distance. The stop array was used to eliminate crosstalk and to achieve low-distortion imaging [12]. However, it was limited to microlens array imaging in the staring field of view with limited far object distance.

Therefore, in this study, the system model with a moving front group and a fixed rear group is selected to realize the imaging of the microlens array in the swing scanning field of view at an infinite object distance; that is, the light from the off-axis object point is converted into parallel light through the microlens array to achieve beam scanning. The matrix formula of paraxial ray tracing is used to describe the source of crosstalk stray light in the MLA system, and the stray light is eliminated as the stop can block the propagation of light. The design results are simulated based on the system performance parameters to verify the correctness of the theory, realize high contrast and high definition imaging of the system, and guarantee a reliable design and processing of the MLA system.

## 2. Generation Mechanism of Crosstalk Stray Light

**Figure 1:**
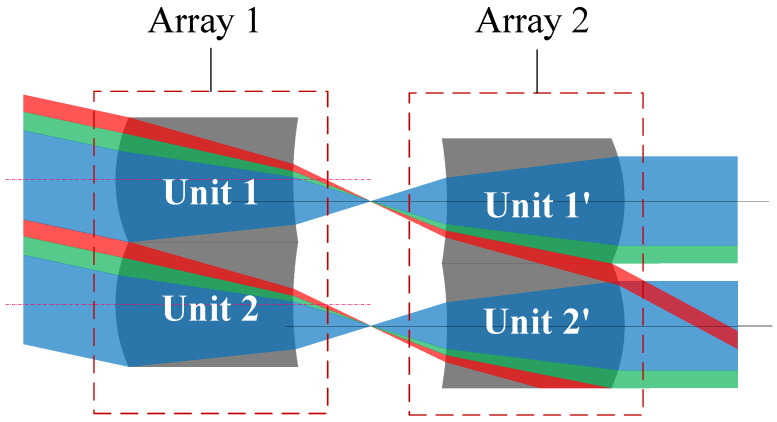
Schematic of the MLA system crosstalk stray light.

The scanning function of the MLA system is realized as a result of the mutual displacement between the front and rear microlens arrays. With increasing the swing angle, the light that should have been emitted from array lens unit 1 enters unit 1′, and some of the light may reach the adjacent array lens unit 2′, interfering with the normal beam imaging of the array lens unit, thus reaching the image plane without following the specified path. At the same time, this part of light causes light loss of the array lens unit 1′, and thus light cannot fill the MLA aperture, as shown in Figure 1.

**Figure 2:**
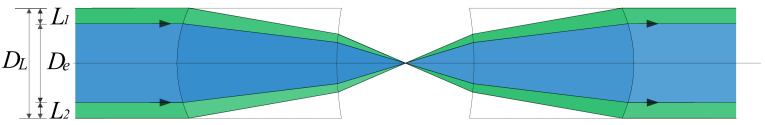
Schematic of zero field-of-view of a single group microlens system.

The scanning function of the microlens array is realized owing to mutual displacement, and the light transmission for different swing angles corresponds to the varying stray light distribution. For the convenience of discussion, we select two microlens symmetrical arrangements with identical positive power for analysis. Figure 2 shows a schematic of a single microlens system constructed using two positive lenses. At this time, it is a zero field of view, that is, the two lenses have no relative displacement.

The entrance pupil of the system is defined as *De*, which is smaller than its array lens aperture *D_L_*. When light enters the full aperture, the blue area represents the imaging light, and the green area represents the light that has still not passed the corresponding array aperture; this light in the green area is defined as Type 1 stray light. The apertures of the light line in the green area above and below *De* are *L*_1_ and *L*_2_, respectively.

**Figure 3:**
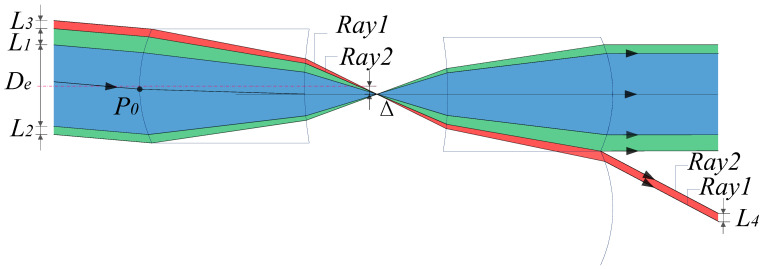
Schematic of MLA system at any swing angle.

When the field angle gradually increases, the displacement distance between the two positive power components of the corresponding MLA system is Δ. The red area represents the light entering the adjacent array aperture, and its incident aperture is defined as *L*_3_. Normal imaging light from other units is superimposed to form an image together on the image plane, reducing the imaging quality; this is defined as Type 2 stray light. At this time, Type 1 stray light below *De* is relatively reduced, but it does not disappear, as shown in Figure 3.

**Figure 4:**
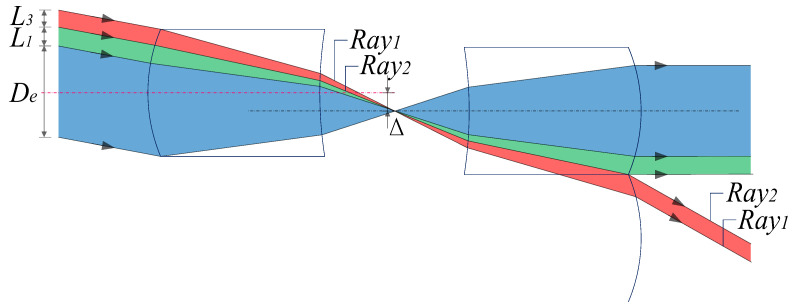
Schematic of the MLA system at the maximum swing angle.

When the field angle continues to increase to the maximum, the lower edge of the imaging light reaches the lower side of the microlens unit, Type 1 stray light under *De* gradually disappears, and Type 2 stray light gradually increases, as shown in Figure 4.

## 3. Theoretical Analysis of Crosstalk Stray Light

**Figure 5:**
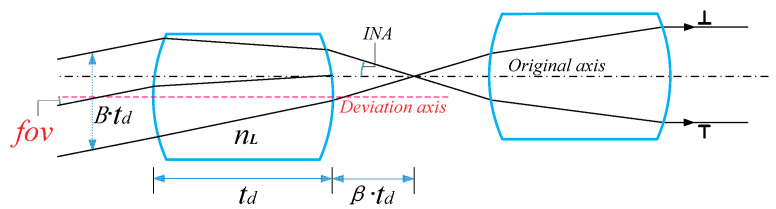
Schematic of MLA system parameters.

This section analyzes the light transmission of the MLA system at any swing angle, as shown in Figure 3. Expressions for various beam apertures (1)–(3) are derived from the perspective of geometric optics, where $P_{0}$ is the intersection of the main ray of the imaging ray and the first surface. At the same time, the schematic of the system parameters is shown in Figure 3 and Figure 5.

(1) $$L_{1}=\frac{D_{L}}{2}-\frac{D_{e}}{2},$$

(2) $$L_{2}=\frac{D_{L}}{2}+(P_{0}-\frac{D_{e}}{2}),$$

(3) $$L_{3}=\frac{D_{L}}{2}-(P_{0}+\frac{D_{L}}{2})=-P_{0}.$$

Here, *fov*, $n_{L}$, *INA*, and *β* represent the angle of view, refractive index of MLA system material, numerical aperture angle between positive power components, and ratio of the back intercept of the power component to the thickness of the lens, respectively. By constructing the relationship between the parameters, the system parameter expressions (4)–(6) can be obtained according to the derivation presented a previous report [13]. $t_{d}$ is the central thickness of the microlens and *B* is a coefficient less than 1.

(4) $$D_{e}=B\cdot t_{d},$$

(5) $$\Delta =\frac{fov\cdot B\cdot t_{d}}{2\cdot INA},$$

(6) $$D_{L}=\frac{2\cdot n_{L}\cdot \beta \cdot INA^{2}+fov_{m}\cdot X}{2\cdot n_{L}\cdot \beta \cdot INA^{2}}\cdot B\cdot t_{d}.$$

The ordinate value of $P_{0}$ is obtained by reverse tracking, as follows:

(7) $$P_{0}=-\frac{B\cdot fov\cdot \left(B\cdot n_{L}-2\cdot INA\right)\cdot t_{d}}{4INA^{2}\cdot n_{L}\cdot \beta },$$

where $X=B\cdot n_{L}-2\cdot INA$, and *fov_m_* in the formula represents the maximum field of view. The system can be well described by these basic composition parameters.

Substituting Formulas (4)–(6) into Formulas (1)–(3), we obtain the following

(8) $$L_{1}=\frac{B\cdot fov_{m}\cdot X\cdot t_{d}}{4INA^{2}\cdot n_{L}\cdot \beta },$$

(9) $$L_{2}=-\frac{B\cdot \left(fov-fov_{m}\right)\cdot \left(B\cdot n_{L}-2\cdot INA\right)\cdot t_{d}}{4INA^{2}\cdot n_{L}\cdot \beta },$$

(10) $$L_{3}=\frac{B\cdot fov\cdot X\cdot t_{d}}{4INA^{2}\cdot n_{L}\cdot \beta },$$

Subsequently, we define the upper and lower edge rays of Type 2 stray light in Figure 4 as Ray 1 and Ray 2, respectively, and trace the rays to obtain the propagation law of Type 2 stray light.

**Figure 6:**
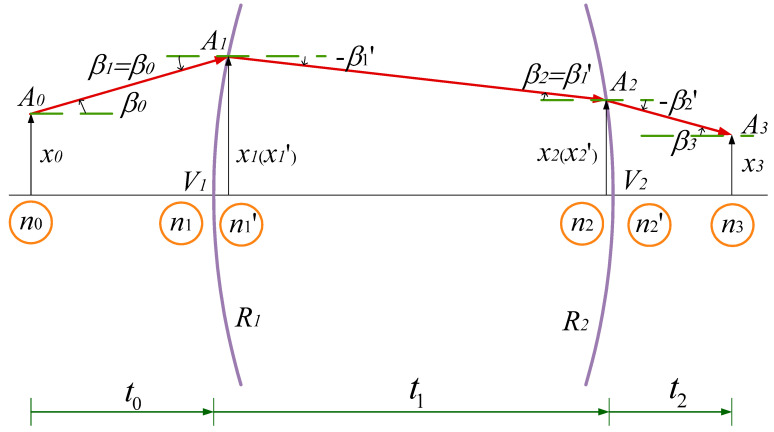
Propagation path diagram of light in single lens.

We discuss the ray tracing matrix representing the system from the point of incidence to the point of exit. <<Optical Imaging and Aberrations>> gives the propagation path diagram of light in a single lens [14], as shown in Figure 6.

Then, the propagation from point $A_{0}$ through the optical system consisting of two refractive surfaces to $A_{3}$ can be described by Equation (11).

(11) $$\left[\begin{array}{c} x_{3}\\ n_{3}\cdot \beta_{3} \end{array}\right]=\left[\begin{array}{cc} 1 & \frac{t_{2}}{{n_{2}}^{\prime }}\\ 0 & 1 \end{array}\right]\cdot \left[\begin{array}{cc} 1 & 0\\ -\frac{{n_{2}}^{\prime }-n_{2}}{R_{2}} & 1 \end{array}\right]\cdot \left[\begin{array}{cc} 1 & \frac{t_{1}}{{n_{1}}^{\prime }}\\ 0 & 1 \end{array}\right]\cdot \left[\begin{array}{cc} 1 & 0\\ -\frac{{n_{1}}^{\prime }-n_{1}}{R_{1}} & 1 \end{array}\right]\cdot \left[\begin{array}{cc} 1 & \frac{t_{0}}{n_{0}}\\ 0 & 1 \end{array}\right]\cdot \left[\begin{array}{c} x_{0}\\ n_{0}\cdot \beta_{0} \end{array}\right]$$

**Figure 7:**
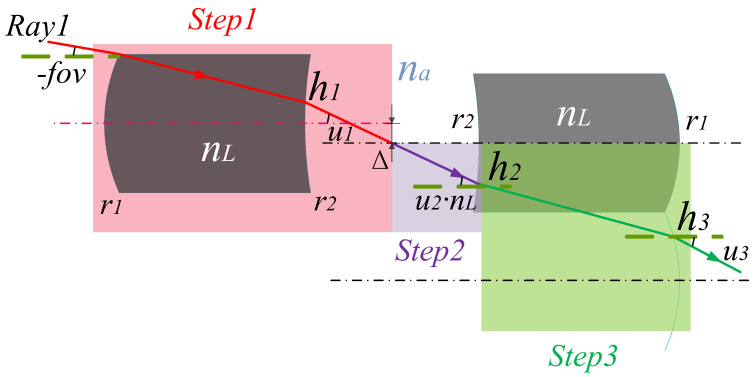
Schematic of the ray-tracing steps of the array system.

Therefore, the microlens array system in this study uses three steps to track, as shown in Figure 7.

Step 1. The matrix is used to trace the light path to the primary image plane. The ray tracing matrix equation is expressed as follows:

(12) $$\left[\begin{array}{c} h_{1}\\ n_{a}\cdot u_{1} \end{array}\right]=\left[\begin{array}{cc} 1 & \frac{t_{b}}{n_{a}}\\ 0 & 1 \end{array}\right]\cdot \left[\begin{array}{cc} 1 & 0\\ \frac{n_{L}-n_{a}}{r_{2}} & 1 \end{array}\right]\cdot \left[\begin{array}{cc} 1 & \frac{t_{d}}{n_{L}}\\ 0 & 1 \end{array}\right]\cdot \left[\begin{array}{cc} 1 & 0\\ \frac{n_{a}-n_{L}}{r_{1}} & 1 \end{array}\right]\cdot \left[\begin{array}{c} \frac{D_{L}}{2}\\ -fov \end{array}\right]$$

Step 2. Trace the optical path from the primary image plane to the inner side of Lens 2. The ray tracing matrix equation is expressed as follows:

(13) $$\left[\begin{array}{c} h_{2}\\ n_{L}\cdot u_{2} \end{array}\right]=\left[\begin{array}{cc} 1 & 0\\ \frac{n_{a}-n_{L}}{-r_{2}} & 1 \end{array}\right]\cdot \left[\begin{array}{cc} 1 & \frac{t_{b}}{n_{a}}\\ 0 & 1 \end{array}\right]\cdot \left[\begin{array}{c} 0\\ n_{a}\cdot u_{1} \end{array}\right]$$

Step 3. Trace the optical path from the inside to the outside of Lens 2. The ray tracing matrix equation is expressed as follows:

(14) $$\left[\begin{array}{c} h_{3}\\ n_{a}\cdot u_{3} \end{array}\right]=\left[\begin{array}{cc} 1 & 0\\ \frac{n_{L}-n_{a}}{-r_{1}} & 1 \end{array}\right]\cdot \left[\begin{array}{cc} 1 & \frac{t_{d}}{n_{L}}\\ 0 & 1 \end{array}\right]\cdot \left[\begin{array}{c} D_{L}+h_{2}\\ n_{L}\cdot u_{2} \end{array}\right]$$

The exit angle $u_{3}$ and height $h_{3}$ of the ray after passing through the array system can be obtained as:

(15) $$u_{3}=-\frac{\left(B-2INA\cdot \beta \right)\cdot \left(fov_{m}\cdot X+2\cdot INA^{2}\cdot n_{L}\cdot \beta \right)}{2\cdot INA^{2}\cdot \beta },$$

(16) $$h_{3}=\frac{2\cdot INA^{2}\cdot n_{L}\cdot \beta -\left(fov-fov_{m}\right)\cdot X}{4\cdot INA^{2}\cdot n_{L}\cdot \beta }\cdot B\cdot t_{d}.$$

Similarly, we can obtain the propagation results of Ray 2 as:

(17) $$u_{4}=-\frac{\left(B-2INA\cdot \beta \right)\cdot \left(fov_{m}\cdot X+2\cdot INA^{2}\cdot n_{L}\cdot \beta \right)}{2\cdot INA^{2}\cdot \beta },$$

(18) $$h_{4}=\frac{\left(fov_{m}\cdot X+2\cdot INA^{2}\cdot n_{L}\cdot \beta \right)}{4\cdot INA^{2}\cdot n_{L}\cdot \beta }\cdot B\cdot t_{d}=\frac{D_{L}}{2}.$$

The exit aperture of Ray 2 can be defined by h4−h3, as shown in Formula (19):

(19) $$L_{4}=\frac{B\cdot fov\cdot t_{d}\cdot X}{4\cdot INA^{2}\cdot n_{L}\cdot \beta }.$$

It can be observed that the sizes of *L*_4_ and *L*_3_ tend to be the same, and the values of *u_4_* and *u_3_* tend to be the same; that is, the angle of the outgoing light of the array system is approximately parallel. This provides an idea for the suppression and elimination of this type of stray light. Therefore, we can select the basic composition parameters of the system to change the system shape, thereby inhibiting the formation of this type of stray light.

## 4. Design of Crosstalk Stray Light Suppression

The generation and theoretical analysis of crosstalk stray light indicate that an optiaml aperture should be selected to effectively suppress stray light.

**Figure 8:**
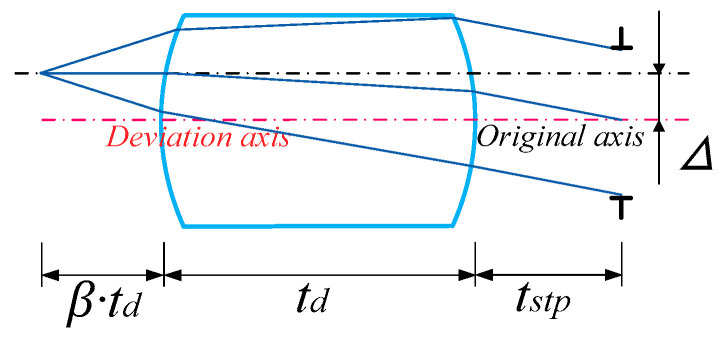
Schematic of the ray-tracing of the array system.

It is assumed that the lenses of the MLA system are symmetrically arranged about the primary image plane with equal power, the central light is parallel to the optical axis at the primary image plane, and the MLA system is usually combined with the infrared optical system for imaging. Therefore, the MLA system has an aperture stop array at the exit pupil, as shown in Figure 8.

The pupillary exit distance *tstp* can be obtained by ray-tracing the positive power lens behind the primary image plane, as shown in Formula (20). To suppress stray light, a double stop array group is set. As shown in Figure 8, Type 1 stray light can be eliminated by setting an aperture stop array; that is, the aperture stop array not only acts as an exit pupil in the system, but also aids in restricting some stray light from participating in imaging. By observing the propagation path of the crosstalk stray light, it is found that residual Type 2 stray light still interferes with imaging. Therefore, we can effectively suppress Type 2 stray light by setting crosstalk suppression stop array and coating the absorption film at any position between the primary image plane and the front surface of the second array of the microlenses.

(20) $$t_{stp}=\frac{\left(B\cdot n_{L}-2INA\right)}{4INA^{2}\cdot n_{L}\cdot \beta }\cdot B\cdot t_{d}.$$

**Figure 9:**
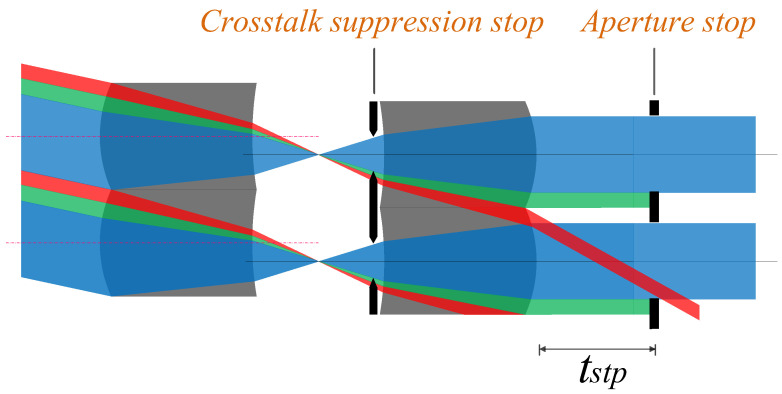
Schematic of the stop placement of an array system.

**Figure 10:**
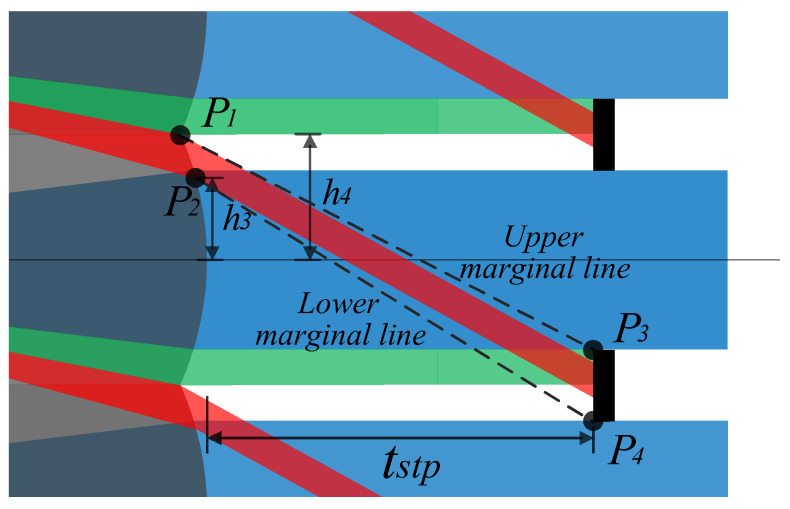
Specific schematic of the stray light suppression.

Figure 9 is a schematic of the stop placement of an array system, and Figure 10 illustrates a special case of stray light suppression shown in Figure 9. At this time, owing to the reasonable setting of various parameters of the system, Types 1 and 2 stray light do not need to be set with the crosstalk suppression stop array, but can be independently suppressed by the aperture stop array. *P*_1_ and *P*_2_ are defined as the highest and lowest positions of Ray 2 on the exit pupil plane, respectively. *P*_3_ and *P*_4_ are the upper and lower edge positions of the stop at the exit pupil position, respectively. The expression is shown in Formula (21).

(21) $$\left\{\begin{array}{c} \begin{array}{c} P_{1}:\left(-t_{stp},h_{4}\right),\\ P_{2}:\left(-t_{stp},h_{3}\right), \end{array}\\ P_{3}:\left(0,-\frac{D_{e}}{2}+N\cdot D_{L}\right),\left(N=0,\pm 1,\pm 2\ldots \ldots \right)\\ P_{4}:\left(0,-D_{L}+\frac{D_{e}}{2}+N\cdot D_{L}\right),\left(N=0,\pm 1,\pm 2\ldots \ldots \right) \end{array}\right..$$

Based on Formula (21), we can obtain the upper and lower edge angles as follows:

(22) $$Upper=\left(2N-1\right)\cdot fov_{m}+\frac{4\cdot \left(N-1\right)\cdot INA^{2}\cdot n_{L}\cdot \beta }{X},\left(N=0,\pm 1,\pm 2\ldots \ldots \right),$$

(23) $$Lower=\left(2N-3\right)\cdot fov_{m}+\frac{4\cdot \left(N-1\right)\cdot INA^{2}\cdot n_{L}\cdot \beta }{X},\left(N=0,\pm 1,\pm 2\ldots \ldots \right).$$

Combining Formulas (22), (23), and (15), we obtain

(24) $$fovm\approx \left[-\frac{2\cdot INA^{2}\cdot n_{L}\cdot \beta \cdot \left[\left(B-2INA\cdot \beta \right)\cdot X+4\cdot INA^{2}\cdot \left(N-1\right)\cdot \beta \right]}{X\cdot \left[\left(B-2INA\cdot \beta \right)\cdot X+4\cdot INA^{2}\cdot \left(N+1\right)\cdot \beta \right]},-\frac{2\cdot INA^{2}\cdot n_{L}\cdot \beta \cdot \left[\left(B-2INA\cdot \beta \right)\cdot X+4\cdot INA^{2}\cdot \left(N-1\right)\cdot \beta \right]}{X\cdot \left[\left(B-2INA\cdot \beta \right)\cdot X+4\cdot INA^{2}\cdot \left(N-3\right)\cdot \beta \right]}\right],\left(N=0,\pm 1,\pm 2\ldots \right).$$

When the *fov_m_* value meets the above parameter requirements, it can ensure that the Type 2 stray light is always shielded by the aperture stop array.

**Figure 11:**
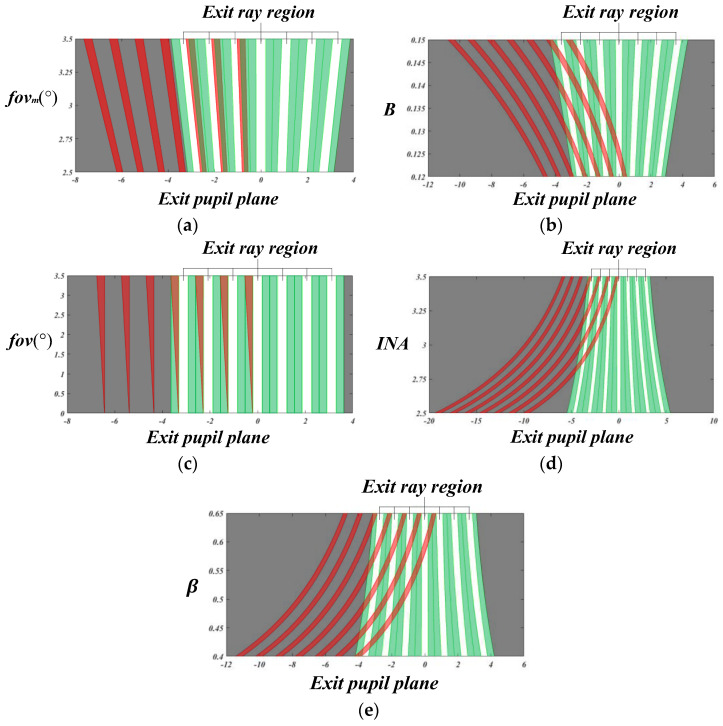
Different plots on the exit pupil plane. (**a**) Plot between *fov_m_* and the exit pupil plane (where *fov = fov_m_*, *B* = 0.1333, *n_L_* = 4.025, *INA* = 3.3438°, *β* = 0.5333, *t_d_* = 3.0). (**b**) Plot between *B* and the exit pupil plane (where *fov_m_* = 3°, *n_L_* = 4.025, *INA* = 3.3438°, *β* = 0.5333, *t_d_* = 3.0). (**c**) Plot between *fov* and the exit pupil plane (where fovm = 3°, *B* = 0.13333, *n_L_* = 4.025, *INA* = 3.3438°, *β* = 0.5333, *t_d_* = 3.0). (**d**) Plot between *INA* and the exit pupil plane (where *fov_m_* = 3°, *B* = 0.13333, *n_L_* = 4.025, *β* =0.5333, *t_d_* = 3.0). (**e**) Plot between *β* and the exit pupil plane (where fovm = 3°, *B*=0.13333, *n_L_* = 4.025, *INA* = 3.3438°, *t_d_* = 3.0).

We discuss the distribution of Type 1 and 2 stray light at the exit pupil position in this section. Figure 11 shows the influence of *fov_m_* parameter changes on the distribution of stray light. Among them, Type 1 stray light, shown in green, can be eliminated through the aperture stop array. The white, red, and black areas represent the exit ray region, Type 2 stray light, and the outside of the array system, respectively. When the red line reaches the black area, Type 2 stray light is emitted outside the array system. When the red line coincides with the green area, Type 2 stray light in this part can be suppressed together through the aperture stop array. When the red line coincides with the white area, Type 2 stray light in this part enters the subsequent system through the exit pupil aperture, causing adverse effects. The design aims to make Type 2 stray light exit the system as far as possible, or make it coincident with Type 1 stray light and be suppressed by the aperture stop array together.

It can be observed from Figure 11a that when the maximum field of view, that is, *fov_m_,* value changes, the aperture range of the system changes, and the aperture range is directly proportional to the *fov_m_* value. At the same time, we observe that the *fov_m_* value has a negligible effect on the change in Type 2 stray light.

It can be observed from Figure 11b that when the B value changes, the aperture range of the system changes, and the aperture range is directly proportional to the *B* value. At the same time, we observe that the *B* value has a significant effect on the change in Type 2 stray light. By increasing the *B* value, Type 2 stray light can be emitted at a larger angle, almost to the outside of the system.

Figure 11c shows the relationship between *fov* and the exit pupil plane. It can be observed that a reasonable basic composition parameter of the system can ensure that Type 2 stray light in the system always lies in the area of Type 1 stray light at the exit pupil plane. Finally, Type 2 stray light can be eliminated through a separate aperture stop array.

It can be observed from Figure 11d that when the numerical value of *INA* changes, the aperture range of the system changes significantly, and the aperture range is inversely proportional to the numerical value of *INA*. By reducing the *INA* value, Type 2 stray light can be emitted at a larger angle, and can be emitted to the outside of the system.

It can be observed from Figure 11e that when the value of *β* changes, the aperture range of the system changes significantly, and the aperture range is inversely proportional to the *β* value. By decreasing the *β* value, Type 2 stray light can be emitted at a larger angle, and this stray light can be emitted to the outside of the system.

We can observe that increasing the *B* value, decreasing the *INA* value, or decreasing the *β* value can realize a large exit angle, allowing Type 2 stray light to exit the system directly. At the same time, the system design parameters can be appropriately selected so that Type 2 stray light whose exit range is still in the system always coincides with the area of Type 1 stray light, and the system crosstalk stray light can be effectively suppressed through the aperture stop array. Thus, this design aims to reduce the influence of stray light as much as possible without additional stops, and to provide guidance for the subsequent system simulation.

## 5. Instance System Verification

Considering a specific infrared band microlens array scanning system as an example, the presented theory is validated. See Table 1 for detailed parameters of the system.

**Figure 12:**
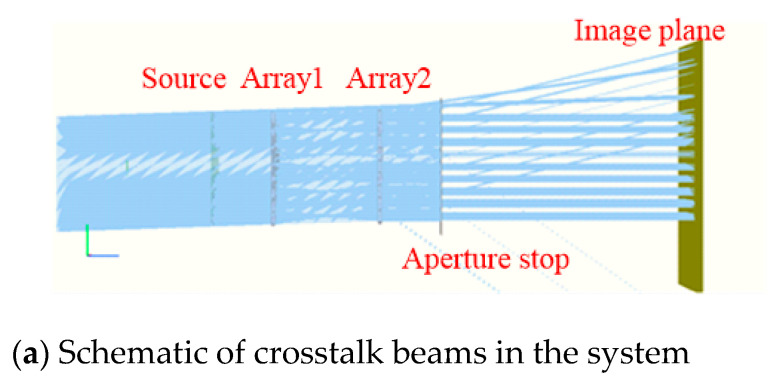

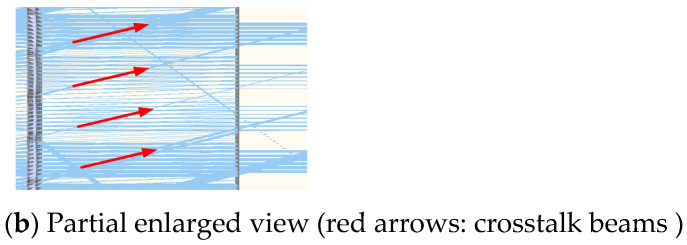
FRED simulation of crosstalk phenomenon in the microlens array.

The optical system is constructed according to the above parameters to obtain the corresponding arrangement of two rows of 9 × 9 microlens arrays. An aperture stop array is placed at the exit pupil. The optical engineering simulation software FRED is used to simulate the crosstalk of the microlens array with only aperture stop array and no crosstalk suppression stop array when the microlens array is staggered along the Y axis and the swing angle is −2°, as shown in Figure 12.

**Figure 13:**
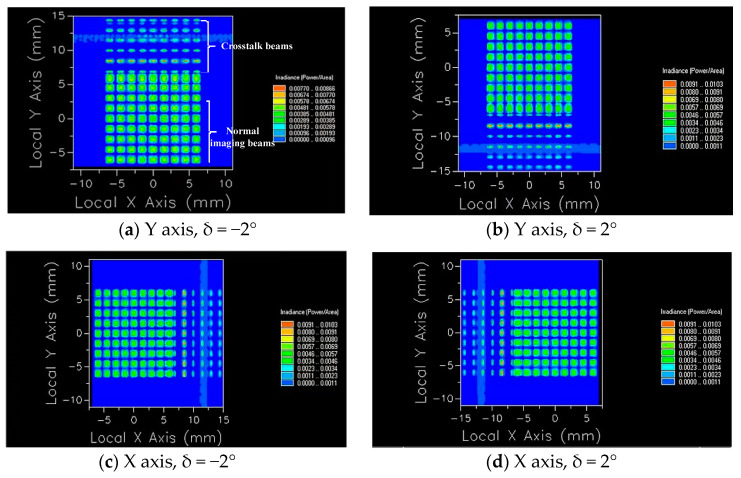
Imaging diagrams under several characteristic situations.

To observe the crosstalk phenomenon clearly, FRED is used to simulate a small amount of oblique parallel beams passing through the microlens array system, and two identical microlens arrays are symmetrically arranged to form a telescope system to ensure parallel beam emission, and we set the light source energy to 1 W. It can be observed from Figure 12 that when the system has only an aperture stop array, the crosstalk beams mentioned in the theoretical analysis do exist in the light transmission process of the system and continue to propagate through the aperture stop array to interfere with imaging. As the lenses are arranged in an array, the crosstalk beams generated by each lens unit are arranged at the same angle, and they exit periodically, and the image power distribution is shown in Figure 13a.

The above simulation shows the crosstalk of the array when it moves relative to the Y axis and δ = −2°. When the image plane is at different positions, the number of lines with energy aliasing will be different. At the same time, because the microlens array is a two-dimensional dynamic scanning, light may enter from any direction, and crosstalk will occur at different positions of the array. As shown in Figure 13, image power distributions on the plane under several characteristic situations are given.

We take the Y axis, δ = −2° as an example. As the normal imaging beam and crosstalk beam propagate on the image plane through the aperture stop array and distribute at intervals, respectively; at this time, we adjust the position of the image plane so that the crosstalk beam can be clearly observed. We use FRED to perform advanced ray tracing, and the beams generate power separately on the image plane and separate from each other, as shown in Figure 13a. Table 2 shows the power values of each aperture in Figure 13a.

**Figure 14:**
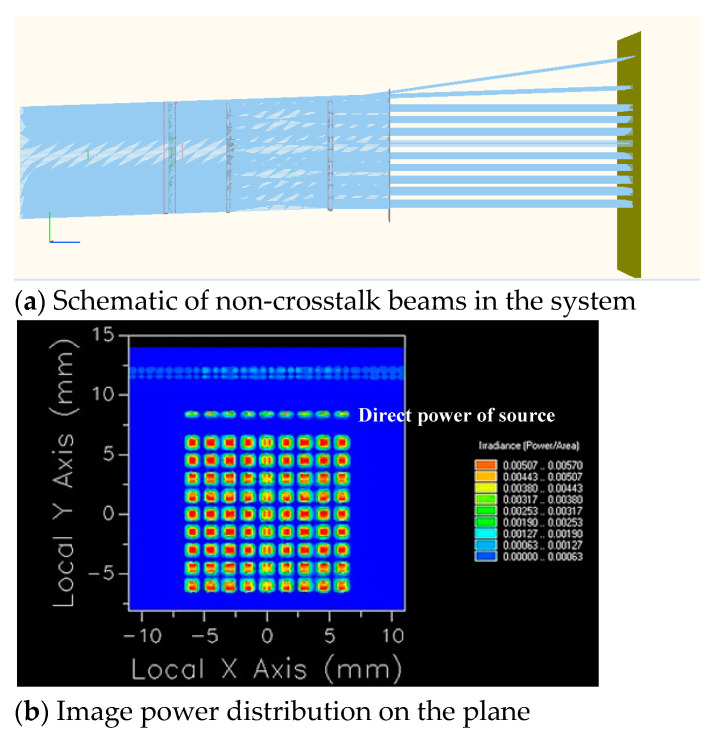
Imaging diagram with a crosstalk suppression stop array (Y axis, δ = −2°).

From the data in Table 2 and Figure 13, it can be observed that the 9 × 9 microlens array system has crosstalk beams superimposed on normal beams, that is, images and energy superimposed on the first and second lines of the array interfere with normal beam imaging. Therefore, we place a crosstalk suppression stop array at a distance of 0.2 mm from the front surface of the second row of microlens arrays to avoid crosstalk beams interfering with imaging in the system. Figure 14 illustrates the simulation of the imaging of the system after adding the crosstalk suppression stop array.

The power values of each aperture are given according to Figure 14, as shown in Table 3.

It can be observed from Figure 14a that there is no crosstalk beam generated after the crosstalk suppression stop array is placed. Figure 14b shows the image power distribution on the plane. The power in the first line of Figure 14b is generated by direct power of the light source, which can be eliminated by adjusting the size of the light source, and it is ignored. In addition, the power corresponding to each aperture is similar to that shown in Table 3, evenly distributed on the image plane, and the crosstalk beam has been effectively suppressed.

**Figure 15:**
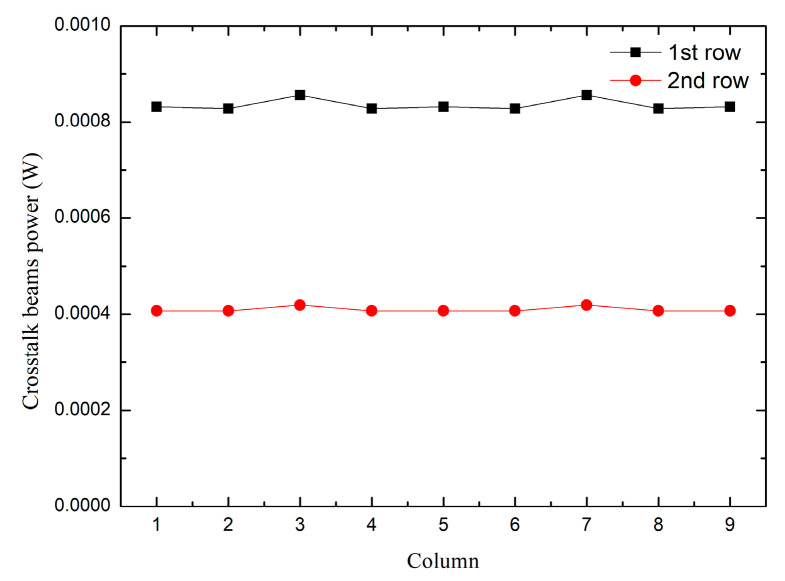
Diagram with crosstalk power values of the two rows (Y axis, δ = −2°).

By comparing the data in Table 2 and Table 3, it can be seen that after the stop array is added, the power of the first and second rows of the array decreases. The reduced value is the crosstalk power superimposed on each aperture. Figure 15 plots the crosstalk power values of the two rows.

As the crosstalk value in the second line is generated by the edge array, the crosstalk value is reduced compared with the first line, which makes the curves of the two lines have a certain distance. Increasing the size of the light source can reduce the distance.

Therefore, the crosstalk suppression stop array can be added according to the actual situation to enable the system to achieve interference-free transmission. Similarly, when the relative displacement of the microlens array corresponds to different swing angles, the crosstalk suppression stop array can also suppress the propagation of the crosstalk beams. In addition, the method of adding crosstalk suppression stop arrays to eliminate crosstalk can be extended to the M * N microlens array.

## 6. Conclusions

This study aimed to solve the basic problem of crosstalk generated by the microlens array system under dynamic scanning and to clarify the mechanism and influence of crosstalk generation. Taking advantage of the characteristics of the stop array that can limit beam transmission, a design method of a double-stop array group was proposed to suppress the crosstalk beams. We analyzed the relationship between the structural parameters of the microlens array and the position and size of the stop array. The matrix ray tracing mode was constructed to complete the system crosstalk stray light model and realize the light control of different incident angles and eccentricity in dynamic scanning. Then, the stray light analysis software FRED was used to simulate an example of a system in different situations and to analyze its stray light suppression effect to verify the correctness of the theoretical model. Furthermore, the microlens array system can enlarge the dynamic scanning range without crosstalk, thus verifying the feasibility of the design method. In addition, it can aid in guiding the design and analysis of the microlens array scanning and searching system in the future.

## Figures and Tables

**Table 1:** Array system parameters.

Parameter	Value
Array size	13.5 mm × 13.5 mm
Number	9 × 9
Single lens size	1.5 mm × 1.5 mm
Layer	2
Central wavelength	4 μm
Lens shape	rectangle
*n*	*n*_1_ = *n*_2_ = 4.025058
Swinging angle δ	4° × 4°

**Table 2:** Power value (Watts) of each aperture (Y axis, δ = −2°).

	1	2	3	4	5	6	7	8	9
1	0.005319	0.005183	0.005211	0.005183	0.005319	0.005183	0.005211	0.005183	0.005319
2	0.004894	0.004762	0.004774	0.004762	0.004894	0.004762	0.004774	0.004762	0.004894
3	0.004623	0.004487	0.004487	0.004487	0.004623	0.004487	0.004487	0.004487	0.004623
4	0.004487	0.004355	0.004355	0.004355	0.004486	0.004355	0.004355	0.004355	0.004487
5	0.004487	0.004355	0.004355	0.004355	0.004486	0.004355	0.004355	0.004355	0.004487
6	0.004487	0.004355	0.004355	0.004355	0.004487	0.004355	0.004355	0.004355	0.004487
7	0.004623	0.004487	0.004487	0.004487	0.004623	0.004487	0.004487	0.004487	0.004623
8	0.004487	0.004355	0.004355	0.004355	0.004487	0.004355	0.004355	0.004355	0.004487
9	0.004487	0.004355	0.004355	0.004355	0.004487	0.004355	0.004355	0.004355	0.004487

**Table 3:** Power value (Watts) of each aperture with crosstalk suppression stop array (Y axis, δ = −2°).

	1	2	3	4	5	6	7	8	9
1	0.004487	0.004355	0.004355	0.004355	0.004487	0.004355	0.004355	0.004355	0.004487
2	0.004487	0.004355	0.004355	0.004355	0.004487	0.004355	0.004355	0.004355	0.004487
3	0.004623	0.004487	0.004487	0.004487	0.004623	0.004487	0.004487	0.004487	0.004623
4	0.004487	0.004355	0.004355	0.004355	0.004486	0.004355	0.004355	0.004355	0.004487
5	0.004487	0.004355	0.004355	0.004355	0.004486	0.004355	0.004355	0.004355	0.004487
6	0.004487	0.004355	0.004355	0.004355	0.004487	0.004355	0.004355	0.004355	0.004487
7	0.004623	0.004487	0.004487	0.004487	0.004623	0.004487	0.004487	0.004487	0.004623
8	0.004487	0.004355	0.004355	0.004355	0.004487	0.004355	0.004355	0.004355	0.004487
9	0.004487	0.004355	0.004355	0.004355	0.004487	0.004355	0.004355	0.004355	0.004487

## Data Availability

Data underlying the results presented in this paper are not publicly available at this time but may be obtained from the authors upon reasonable request.
